# Cholesterol metabolism in innate and adaptive response

**DOI:** 10.12688/f1000research.15500.1

**Published:** 2018-10-16

**Authors:** Andrea Reboldi, Eric Dang

**Affiliations:** 1 Department of Pathology, University of Massachussetts Medical School, Worcester, Massachusetts, 01605, USA; 2 Department of Biochemistry and Biophysics, University of California, San Francisco, California, 94158, USA

**Keywords:** cholesterol, liver x receptor, sterol response element-binding proteins

## Abstract

It has been long recognized that cholesterol is a critical molecule in mammalian cell biology, primarily for its contribution to the plasma membrane’s composition and its role in assuring proper transmembrane receptor signaling as part of lipid rafts. Efforts have also been made to characterize the cholesterol biosynthetic pathway, cholesterol homeostasis, and cholesterol-derived metabolites in order to gain insights into their dysregulation during metabolic diseases. Despite the central role cholesterol metabolism plays in shaping human health, its regulation during immune activation, such as immune response to pathogens or autoimmune/autoinflammatory diseases, is poorly understood.

The immune system is composed of several type of cells with distinct developmental origin, life span, molecular requirements, and gene expressions. It is unclear whether the same array of cholesterol metabolism regulators are equally employed by different immune cells and whether distinct cholesterol metabolites have similar biological consequences in different immune cells.

In this review, we will describe how cholesterol metabolism is controlled during the adaptive and the innate immune response and the role for intracellular and extracellular receptors for cholesterol and its derivatives.

## Introduction

Cholesterol has a central and ubiquitous role in mammalian cells, as the pathway of cholesterol biosynthesis from acetyl coenzyme A is active in all nucleated cells and cholesterol is an essential component of mammalian plasma membrane, accounting for up to 25% of all membrane lipid. The cholesterol structure confers rigidity to the plasma membrane; thus, a different percentage of cholesterol results in different plasma membrane rigidity. Several biological processes are controlled by cholesterol-containing lipid rafts on the plasma membrane, including transmembrane receptor signaling and virus entry and budding ^1^.

Despite the critical role for cholesterol in mammalian membrane homeostasis, excess cellular cholesterol is toxic and therefore cholesterol biosynthesis requires tight regulation. Cholesterol has been recognized to exert a negative feedback loop on its own biosynthesis and uptake, and its metabolism has been extremely well characterized molecularly. Although virtually all mammalian cells are dependent on cholesterol metabolism, there is a limited understanding of how cholesterol metabolism is regulated in the steady state and during immune activation, such as in response to infection and during autoimmunity.

Dissecting how cholesterol shapes the immune response is complicated by the multilayered, complex role of cholesterol: in addition to having its own effects on cellular homeostasis, cholesterol is the substrate for the production of several metabolites, including oxysterols ^2^, bile acids ^3^, and steroid hormones ^4^. These cholesterol-derived metabolites have diverse immunomodulatory as well as metabolic effects; the intracellular and extracellular receptors for cholesterol and its derivatives have not been completely characterized in every immune cell type; and the potentially different impact of cholesterol origin (dietary or endogenous) on immune activation has not been fully understood. In this review, we will describe the main regulators of cholesterol metabolism and their role in the adaptive and the innate immune response.

## SREBP

Groundbreaking work from Goldstein *et al*. defined cholesterol as a key molecule in suppressing its own biosynthesis through a negative feedback circuit ^5^. Cholesterol exerts its function by preventing sterol response element-binding proteins (SREBPs) from promoting sterol biosynthesis. SREBPs are transcription factors that promote the transcription of enzymes in the cholesterol biosynthetic pathway, including 3-hydroxy-3-methylglutaryl-CoA reductase (HMGCR), as well as the low-density lipoprotein (LDL) receptor, which is responsible for cholesterol uptake ^6^. In the presence of cholesterol, SREBPs are kept in the endoplasmic reticulum (ER) by the multi-transmembrane SREBP cleavage-activating protein (SCAP) which binds the ER-resident insulin-induced gene (INSIG) ^7^. SCAP contains a sterol-sensing domain that is thought to bind ER membrane cholesterol ^8^; when cholesterol levels drop in the ER, SCAP detaches from INSIG through a conformational change ^9^. SCAP then escorts SREBPs into the Golgi, where site 1 and site 2 proteases cleave SREBPs and activate them as transcription factors ^10^. SREBP family members include three proteins: SREBP1a, SREBP1c, and SREBP2 ^11^.

SREBP2 is encoded by the *Srebf2* gene, whereas SREBP-1a and SREBP-1c both are encoded by the *Srebf1* gene: SREBP-1a and SREBP-1c are different isoforms of SREBP1 that arise from alternate promoter usage. Despite their similar regulation, SREBP proteins have a distinct effect on cellular lipid metabolism. SREBP1c controls the transcription of genes involved in the biosynthesis of fatty acids; SREBP2 controls the transcription of genes involved in cholesterol biosynthesis, intracellular lipid movement, and lipoprotein import, whereas the SREBP1a transcriptional regulon partially overlaps with that of SREBP1c and SREBP2.

## LXR

The liver X receptor alpha (LXRα) and beta (LXRβ) are members of the nuclear hormone receptor family of transcription factors that have key roles in regulating the homeostasis of cholesterol and fatty acids. LXRα and LXRβ have distinct patterns of expression; LXRβ is ubiquitously expressed across most cell types, whereas LXRα is restricted mainly to adipose tissue, the liver, and the intestine.

LXRs control intracellular levels of cholesterol by transcribing genes that encode proteins involved in sterol efflux, such as *Abca1*, *Abcg1*, and *Apoe*, therefore preventing cholesterol accumulation. LXRs also induce the expression of SREBP1c and enzymes involved in fatty acid remodeling, such as *Elovl5*, *Fads2*, *Scd1*, and *Scd2*. As LXRs are thought to work in a ligand-dependent manner, several efforts have been made to define the endogenous ligands for LXRs, but so far the identity of native ligands that activate LXRs *in vivo* has remained elusive.

## Oxysterols

Cholesterol can be enzymatically modified to form metabolites with diverse bioactivities ^3^. The most characterized proximal cholesterol metabolites are oxysterols, which are oxidized via hydroxylation reactions typically on the non-cyclic side chain of cholesterol. 25-hydroxycholesterol (25-HC) is synthesized from cholesterol by the addition of a hydroxyl group at position 25: the enzyme responsible for 25-hydroxylation reaction is cholesterol 25-hydroxylase (CH25H), a multi-transmembrane ER protein. 27-HC is instead generated through the action of the sterol 27-hydroxylase CYP27A1, a mitochondrial cytochrome P450 oxidase. Both 25-HC and 27-HC can be hydroxylated at the 7α position by the enzyme 7α-hydroxylase (CYP7B1) to generate 7α,25-HC and 7α,27-HC. CYP7B1 is a cytochrome P450 family enzyme and is also situated in the ER. Both CYP7B1 and CYP27A1 are abundant in the liver, whereas CH25H is virtually absent in the liver under homeostatic conditions. This is in line with the described role of CYP27A1 and CYP7B1, but not CH25H, in the conversion of cholesterol into bile acids, making several oxysterols *de facto* bile acid synthesis intermediates. Interestingly, all three enzymes can be found, albeit with a range of expression, in several other tissues. These include primary and secondary lymphoid organs, suggesting that they might play a role in a distinct set of biological processes, particularly immune regulation. Similar to other 7α-hydroxylated sterols, 7α,25-HC and 7α,27-HC are metabolized by the enzyme 3β-hydroxysteroid dehydrogenase type 7 (HSD3B7), which is situated in the ER. HSD3B7, similar to CYP7B1 and CYP27A1, is very abundant in the liver and it is essential for bile acid synthesis; nevertheless, its ability to metabolize 7α,25-HC and its expression in other tissues suggest that it has an extrahepatic role in controlling oxysterol levels.

## Regulation of the SREBP pathway in macrophages

Studies from the 1970s found that the addition of 25-HC to mitogen-stimulated lymphocyte cultures could repress proliferation ^12^. Intriguingly, the inhibited lymphocyte proliferation in the presence of 25-HC could be rescued by cholesterol, suggesting that 25-HC could repress cholesterol biosynthesis. This phenomenon was subsequently explained by studies demonstrating that 25-HC can inhibit SREBP activation via direct binding to Insig-1 ^9^. This causes Insig-1 to associate with the SCAP/SREBP complex and trap it in the ER. These data suggest that there are two layers of feedback regulation for the SREBP pathway: cholesterol-based feedback and oxysterol-based feedback.

Despite ample biochemical evidence that 25-HC is a potent inhibitor of SREBP, the role of oxysterols in controlling cholesterol biosynthesis has been controversial. Initial analysis of Ch25h-knockout mice, which are thus 25-HC-deficient, found no evidence of homeostatic cholesterol abnormalities ^3^. This suggested that perhaps endogenous 25-HC plays a non-essential or redundant role in restricting SREBP activity. However, it is important to consider that levels of homeostatic cholesterol, particularly serum LDL and high-density lipoprotein (HDL), are controlled predominantly by hepatic lipoprotein metabolism. As described above, hepatic *Ch25h* expression levels are very low at baseline and this likely explains the lack of a genetic requirement for Ch25h in controlling whole animal cholesterol metabolism.

It is now appreciated that Ch25h is a type I interferon (IFN-I)-inducible enzyme, particularly in myeloid cells ^3– 5^. This is consistent with previous studies showing that IFN-I causes repression of cholesterol biosynthesis ^13^. However, Blanc *et al*. and Liu *et al*. found that while Ch25h/25-HC has antiviral activity downstream of IFN-I signaling, this was uncoupled from its ability to inhibit the SREBP pathway, as expression of the constitutively active nuclear form of SREBP2 could not rescue the antiviral activity ^13, 14^. The authors proposed distinct models to explain the antiviral action of 25-HC. Liu *et al*. found that 25-HC inhibits the entry of viruses into cells, potentially through remodeling of the host cell plasma membrane to prevent viral particle fusion. On the other hand, Blanc *et al*. suggested that 25-HC blocks viral replication through inhibition of isoprenoid biosynthesis, as the addition of geranylgeraniol to 25-HC-treated cells could rescue viral titers. Recently, Bensinger *et al*. uncovered an additional co-regulation between IFN-I and cholesterol, as they showed that limiting the pool size of cholesterol synthesis induces type I IFN response in a STING (stimulator of type I IFN genes)-dependent manner and protects them from viral infection ^15^. Similarly, Ghazal *et al*. showed that an IFN-induced microRNA (miRNA), miR-342-5p, targets mevalonate-sterol biosynthesis through multiple mechanisms suppressing the pathway at different functional levels, further underlining the importance of IFN-I-cholesterol biosynthetic cross-talk in antiviral response ^16^.

While the studies on IFN-I induction of Ch25h in the context of viral infection did point to a physiological role of oxysterols in controlling cholesterol metabolism, Reboldi *et al*. demonstrated for the first time that endogenous 25-HC controls SREBP activity in the context of inflammation ^17^. The authors found that Ch25h knockout mice have increased interleukin-17A (IL-17A) production from αβ and γδ T cells in secondary lymphoid organs. Additionally, bone marrow-derived macrophages (BMDMs) from Ch25h-knockout mice have augmented transcription and secretion of the cytokine IL-1β, a potent inducer of IL-17A from lymphocytes, in response to lipopolysaccharide (LPS). Importantly, transcriptome analysis revealed that while unstimulated Ch25h-knockout BMDMs have no difference in SREBP pathway activity compared with wild-type cells, Ch25h-deficient BMDMs show hyper-SREBP activity upon LPS stimulation. Overexpression of Insig-1 and deletion of SCAP both decreased Il1b mRNA levels, suggesting that SREBP can promote Il1b transcription either directly or indirectly.

IL-1β and its family member IL-18 are unique among cytokines in that they lack a leader sequence for canonical protein secretion ^18^. Instead, IL-1β and IL-18 normally exist as cytosolic pro-form cytokines that must be cleaved for activation and cellular release. Activation of these cytokines is regulated by a multimeric protein complex known as “the inflammasome”, which consists of an NLR/ALR family sensor protein, an adaptor protein called ASC, and the cysteine protease caspase-1. Ligand binding to an inflammasome sensor protein causes ATP-dependent oligomerization and recruitment of ASC through PYRIN domain interactions. ASC then recruits caspase-1 via binding of their respective CARD domains. Because this complex is oligomeric, multiple caspase-1 proteins are brought into close proximity, which promotes caspase-1 autoproteolysis and release of its active form that subsequently can process IL-1β and IL-18.

Since Ch25h-deficient BMDMs hyper-secrete IL-1β in response to LPS, this leads to the question of how the SREBP pathway and cholesterol pathway connect with inflammasome activation. Dang *et al*. found that dysregulation of macrophage cholesterol biosynthesis is sufficient to promote inflammasome activation and IL-1β processing ^19^. Surprisingly, this seems to be downstream of the AIM2 inflammasome sensor protein, which canonically recognizes cytosolic double-stranded DNA (dsDNA). The authors found that dysregulated cholesterol biosynthesis in Ch25h-deficient BMDMs can cause mitochondrial damage, leading to release of mitochondrial DNA into the cytosol, thus providing a spurious ligand for AIM2. Additionally, NLRP3 was found to play a partially redundant role in driving inflammasome activation in response to cholesterol. This is consistent with studies showing that macrophage cholesterol accumulation in Abca1/Abcg1 double-knockout mice causes NLRP3 activation ^20^.

## Liver X receptors control macrophage inflammatory signaling

As described above, LXRs are key transcriptional regulators of cholesterol efflux in response to sterol overload. However, like SREBPs, LXRs are appreciated to have additional roles in controlling macrophage inflammation. Transcriptional studies found that the addition of the synthetic LXR agonist GW3965 to BMDMs could repress the expression of nuclear factor-kappa B (NF-κB) target genes in response to LPS treatment ^21^. This was shown to be dependent on LXRs, as LXRαβ double-knockout macrophages were no longer responsive to GW3965, clearly demonstrating that LXR has the capacity to inhibit inflammation. Less clear is whether inflammatory/anti-inflammatory signals promote the expression of endogenous LXR agonists in macrophages, as most of the functional studies looking at the role of LXRs in inflammation have relied on the use of synthetic agonists.

The mechanism underlying LXR-dependent suppression of inflammatory signaling is also open to debate. Current evidence suggests that ligand binding promotes LXR SUMOylation, which causes LXR to target gene promoters for Toll-like receptor (TLR) target genes ^22^. NCoR co-repressor complexes need to be removed from gene promoters in order for transcriptional activation to occur. This removal requires a member of the NCoR complex known as Coronin 2A, which binds to nuclear actin and thus promotes actin-dependent removal of NCoR from gene promoters ^23^. It has been shown that SUMOylated LXR prevents turnover of NCoR complexes from TLR target gene promoters by interfering with Coronin 2A binding to nuclear actin ^23^. These data suggest a direct inhibition model for LXR repression of inflammatory gene induction, whereby binding to specific gene promoters prevents their transcription. However, a study by Ito *et al*. suggested that LXR primarily inhibits inflammatory signaling via its ability to promote Abca1/Abcg1-dependent cholesterol efflux ^24^. The authors found that GW3965 is no longer capable of suppressing TLR target gene transcription in Abca1-knockout macrophages that are impaired for efficient cholesterol efflux. The authors argue that the promotion of cholesterol efflux by LXR prevents the recruitment of MyD88 and TRAF6 to TLR signaling clusters on the plasma membrane ^24^. Rong *et al*. additionally found that treatment of macrophages with GW3965 promotes the transcription of Lpcat3 in an LXR-dependent manner ^25^. Lpcat3 is a phospholipid (PL) remodeling enzyme that preferentially incorporates polyunsaturated fatty acids into PLs. The authors showed that knockdown of Lpcat3 induces ER stress and activation of NF-κB-dependent cytokines in response to saturated fatty acid challenge. These data suggest that an additionally anti-inflammatory mechanism for LXR is preventing ER stress via induction of Lpcat3. The studies by Ito *et al*. and Rong *et al*. challenge the concept that LXR has direct repressor activity on inflammatory genes. Resolution of these conflicting models will require better reagents to assess genome-wide LXR target binding, as it would be of interest to determine whether Abca1 deficiency affects LXR target choice.

Despite its described role in repressing inflammatory gene induction, LXR has also been described to be required for host survival of intracellular bacterial infection. LXRαβ double-knockout mice are more susceptible to *Listeria monocytogenes* infection, by both survival and colony-forming unit (CFU) analysis ^26^. This was initially argued to be a result of LXR-dependent control of macrophage survival, as LXR-deficient BMDMs were more apoptotic when challenged with bacteria, potentially due to decreased expression of SPα. However, these interpretations are complicated by recent data showing that LXRα is critical for development and maintenance of liver and splenic tissue macrophage populations ^27^. LXRα-knockout mice have dramatically decreased homeostatic numbers of Kupffer cells and splenic marginal zone macrophages, which could be a simple explanation for why these mice are susceptible to bacterial infections with a predilection for those organs. It is not clear whether LXRα promotes the differentiation or survival of liver and spleen macrophage populations. It is tempting to speculate that these macrophages are constantly being loaded with cholesterol because of phagocytosis of red blood cells and other circulating apoptotic cells and thus that the requirement for LXRα reflects an adaptation to tissue-specific metabolic stress.

## Cholesterol metabolism in T-cell and B-cell proliferation

The role of cholesterol in adaptive immune response was originally investigated in the context of lipid raft and antigen receptor signaling. Lipid rafts are plasma membrane microdomains with distinct lipid composition from the surrounding membrane, as they are enriched in cholesterol as well as in glycosphingolipids and sphingomyelin. Lipid rafts are stabilized by the addition of cholesterol, suggesting that intracellular cholesterol metabolism could control raft formation. Historically, the investigation of cholesterol significance in lipid raft during adaptive cell activation relied on chemical compounds, such as methyl β-cyclodextrin, to deplete cholesterol from the membrane raft and then assess B-cell receptor (BCR) and T-cell receptor (TCR) signaling ^28, 29^. However, these molecules often have a global effect on the plasma membrane and actin cytoskeleton, preventing us from drawing a conclusion about the role of cholesterol in the lipid raft. Most recently, the role of cholesterol and its metabolism in TCR signaling has been addressed in a more mechanistic way. For example, Xu *et al*. showed that inhibiting cholesterol esterification in T cells by genetic ablation or pharmacological inhibition of ACAT1, a key cholesterol esterification enzyme, potentiates CD8 ^+^ T-cell effector function against tumors ^30^. This effect was due to augmented plasma membrane cholesterol content that led to increased TCR clustering and immunological synapse formation ^30^. The central role of cholesterol metabolism in controlling TCR signaling has also been highlighted by the findings of Davis *et al*., who showed that cholesterol sulfate, a naturally occurring analog of cholesterol, acts as a negative regulator of TCR signaling by disrupting TCR nanoclusters ^31^.

Upon antigen receptor stimulation, T cells and B cells activate a proliferative program that requires cell enlargement, organelle biogenesis, and cellular replication: in order to sustain the increased metabolic demands, anabolic pathways must be turned on, and they dominate the cellular metabolism ^32^. The first evidence that cholesterol metabolism was critical for lymphocyte activation came from experiments taking advantage of statins, drugs that block HMG-CoA reductase, the enzyme responsible for catalysis in the rate-limiting step of cholesterol biosynthesis. T cells activated *in vitro* and treated with statin showed impaired proliferation ^33^; B cells showed a similar sensitivity to statin, but they strongly upregulate enzymes involved in the cholesterol biosynthetic activation upon CD40–CD40L interaction, the second signal in B-cell activation that has different kinetics and anatomic requirements in comparison with the BCR ligation ^34^. The statin inhibitor effect is related to the cholesterol biosynthetic pathway but might not be directly dependent on the intracellular cholesterol levels. To fully characterize the role of cholesterol metabolism, new genetic and biochemical tools that allow a restricted and timely inhibition of distinct protein in the cholesterol pathway will be needed.

One critical observation of the central role for cholesterol metabolism during T-cell and B-cell proliferation came from the age-dependent expansion of T cells and B cells in mice deficient in both LXRα and LXRβ ^35^. Bensinger *et al*. suggested that oxysterols, not cholesterol per se, were acting as ligands for LXRs, as the addition of exogenous oxysterol activated LXRs in lymphocytes and blocked proliferation in an LXR-dependent fashion.

This observation was in line with the nature of several ligands of other nuclear hormone receptors, which are often small lipophilic molecules, and with the strong upregulation of the enzyme SULT2B1 upon T-cell activation. As SULT2B1 metabolizes oxysterols, it reduces oxysterol intracellular concentration and therefore their availability as LXR ligands.

The decreased signaling through LXR during T-cell activation was accompanied by a concomitant activation of SRBEP pathways, as SREBP-2 and SREBP1 target genes were strongly induced. More recently, it has been shown that, in CD8 ^+^ T cells, both SREBP1a and SREBP2 directly control cell growth and proliferation by mediating the lipid-anabolic program ^36^. T cells lacking SREBP chaperone SCAP showed impaired proliferation and reduced cell enlargement because of a block in G _0_–G _1_. Pathway analysis suggested that SREBPs specifically regulated lipid anabolism and growth of T cells without perturbing TCR signaling or influencing other aspects of T-cell activation.

The described model that places LXR and SREBPs as central players during lymphocyte activation through the intracellular level of cholesterol and oxysterol does not completely recapitulate the *in vivo* observations. For example, although SCAP is critical in inhibiting SREBP activity in T cells and in influencing CD8 ^+^ proliferation *in vitro*, deletion of SCAP *in vivo* does not affect T-cell homeostasis, suggesting the possibility that other pathways are involved in controlling T-cell activation and play a more central role. Lymphocyte proliferation *in vitro* can be reduced in an LXR-dependent way with exogenous oxysterol treatment; nevertheless, mice deficient in the enzymes required for the generation of LXR-activating oxysterols did not show lymphocyte expansion. These discrepancies could have several non-mutually exclusive explanations: it is possible that *in vivo* different oxysterols play a redundant role in controlling lymphocytes through LXR; thus, only concomitant ablation of several enzymes would recapitulate the phenotype observed in LXR-deficient mice. It is also conceivable that other ligands for LXRs exist *in vivo* and that they are not cholesterol derivatives.

## Oxysterols control immune cell migration

Despite the inconclusive data on the role of oxysterols in regulating T-cell and B-cell activation *in vivo* through SREBPs and LXR *in vivo*, more robust evidence exists for the oxysterols as migratory cues that shape the adaptive immune response ^37^. In the effort to identify the ligand for the orphan receptor EBI2, two oxysterols—7α,25-HC and 7α,27-HC—were identified as EBI2 ligands ^38^. 25-HC and 27-HC, which lack a single hydroxyl group and thus are intermediates in the enzymatic reaction that produces 7α,25-HC and 7α,27-HC, are not able to activate EBI2. EBI2 is expressed by several immune cells but is especially high in B cells and is rapidly upregulated upon BCR stimulation. B cells rely on EBI2 to reach the outer follicles shortly after antigen engagement and then use both CCR7 and EBI2 to position at the T–B border and finally in the interfollicular regions and the outer follicle ^39^. B cells deficient in EBI2 failed to properly migrate in the required microanatomic location of lymphoid organs and show reduced T cell-dependent antibody responses ^40, 41^. Genetic manipulation of the enzymes involved in the generation of EBI2 ligands reduced *in vivo* production of 7α,25-HC and 7α,27-HC and phenocopied the EBI2-deficient mice ^42^. In addition to controlling B cells, EBI2 ligands control the positioning of a variety of immune cells *in vivo*: T follicular helper cells ^43, 44^, ILC3 ^45, 46^, and dendritic cells ^47, 48^. *In vivo*, the location of the enzymes required for EBI2 ligand gradient generation has been characterized at least in the spleen, and both hematopoietic and non-hematopoietic cells have been shown to be involved in EBI2 ligand generation ^42^. Ch25h transcript is abundantly found in the outer follicle and within interfollicular regions, including in the marginal zone bridging channel, but is low in B-cell follicles and the T-cell zone. In contrast, Cyp27a1 transcript is abundant in marginal zone bridging channels as well as in the T-cell zone but low in B-cell follicles. The transcript for Cyp7b1, the enzyme that can metabolize both 25-HC and 27-HC to the corresponding 7a EBI2 ligand, is instead more evenly distributed ^49^. Such striking differences for the splenic pattern of Ch25h and Cyp27a1 suggest that, although both act on EBI2, 7α,25-HC and 7α,27-HC might be sensed by distinct cells: why multiple EBI2 ligands exist and what their functional implications are *in vivo* are not fully understood.

## Cholesterol biosynthetic intermediates as RORγt ligands

Oxysterols, including 25-HC, have been initially identified as RAR-related orphan receptor gamma t (RORγt) activators in biochemical assays ^50^. RORγt is an orphan nuclear receptor that is critical for lymphoid tissue organogenesis, T helper 17 (Th17) cells, and innate lymphoid cell group 3: digoxin was identified as an RORγt antagonist on the basis of its ability to displace 25-HC binding to RORγt ^51^. However, mice lacking 25-HC production did not show decreased IL-17 response or impaired lymphoid organogenesis, ruling out 25-HC as an RORγt activator *in vivo*. Two other oxysterols—7b,27-HC and 7a,27-HC—have also been suggested to be natural ligands for RORγt ^52^, and mice deficient in the enzyme Cyp27a1 indeed showed reduced IL-17-producing cells. Such reduction was observed only in young animals, and Cyp27a1-deficient mice were not protected during IL-17-dependent immune disease models, suggesting that other RORγt ligands—endogenous or exogenous or both—might exist.

More recently, RORγt ligand was mapped as a cholesterol biosynthetic intermediate (CBI) by an insect cell-based RORγt reporter system ^53^. Deficiency in two enzymes involved in CBI resulted in distinct phenotypes for RORγt+ cells: lack of Cyp51 showed smaller lymph node anlagen in embryo at E14.5, while T cell-restricted Sc4mol deficiency led to partial reduction of *in vitro* Th17 cells but had no impact on lymph node development. Such partial and cell-specific phenotypes suggest that multiple ligands could regulate RORγt function *in vivo* and specific CBI might act as an RORγt ligand in distinct cells.

More studies are needed to fully capture the *in vivo* production of cholesterol metabolites and their source and their effect on immune cells. Only the systematic combination of enzymatic deficiency and receptor deficiency *in vivo* will allow us to dissect the role of the cholesterol metabolite in the maintenance, regulation, and activation of both innate and adaptive immune systems.
